# Wide Spectrum Analysis of Human Papillomavirus Genotypes in External Anogenital Warts

**DOI:** 10.3390/vaccines9060604

**Published:** 2021-06-05

**Authors:** Orsolya Rideg, Angéla Oszter, Evelin Makk, Endre Kálmán, Kornélia Farkas, Tamás Tornóczky, Krisztina Kovács

**Affiliations:** 1 Department of Pathology, Medical School and Clinical Center, University of Pécs, 7624 Pécs, Hungary; rideg.orsolya@pte.hu (O.R.); angela.oszter@pte.hu (A.O.); makk.evelin@pte.hu (E.M.); kalman.endre@pte.hu (E.K.); kovacs.krisztina2@pte.hu (K.K.); 2 Department of Medical Imaging, Medical School and Clinical Center, University of Pécs, 7624 Pécs, Hungary; 3 Institute of Bioanalysis, Medical School and Clinical Center, University of Pécs, 7624 Pécs, Hungary; farkas.kornelia@pte.hu

**Keywords:** external anogenital warts, prevalence, multi-infection, HPV genotyping, HPV Direct Flow CHIP

## Abstract

External anogenital warts (EGW) are primarily associated with the low-risk human papillomavirus (HPV) genotypes 6 and 11, though coinfection with other low-risk and oncogenic high-risk HPV genotypes also occurs. Although there have been many studies on HPV-associated disease, the prevalence of HPV genotypes associated with EGW is not well characterized. The objective of our retrospective study was to determine the prevalence of HPV genotypes among patients diagnosed with EGW in the south-west of Hungary. Archived formalin-fixed paraffin-embedded tissues from 94 patients were processed in our study. HPV genotypes were determined, applying HPV Direct Flow CHIP test. The overall prevalence of HPV DNA in the EGW samples was 100%, yielding 131 infections among the 94 samples. Of these cases, 72.3% were mono while 27.6% were multi-infections. Out of the 131 infections, the cumulative prevalence of HPV 6 and 11 was 71%. A total of 98.9% of the samples were carrying at least one of these genotypes, while 19.1% of the cases occurred with at least one high-risk genotype. Data from our study could provide invaluable information concerning the prevalence of HPV types among patients with EGW, enabling improved assessment of the actual and future efficacy of vaccination programs, vaccine development, and forecast changes in infection patterns.

## 1. Background

**Figure 1:**
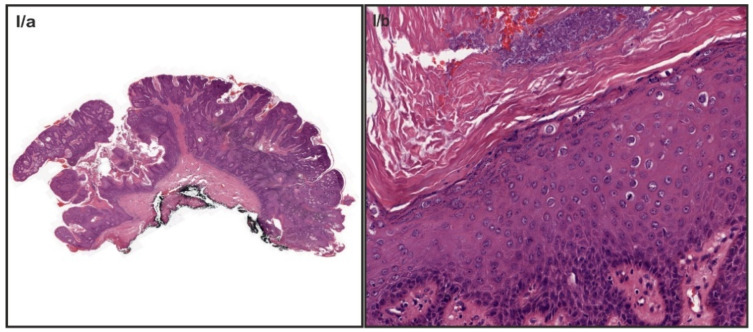
Histological characteristics of external genital warts. Note, the cauliflower-like architecture at the low power (**I/a**), Acanthosis, and parakeratosis with the presence of koilocytes in mid power image (**I/b**).

Human papillomavirus (HPV) infection is responsible for the development of external anogenital warts (EGW), often referred to as external condyloma acuminata (Figure 1). External anogenital warts rank among the most frequent sexually transmitted diseases, approximately 65% of people who have sex with an infected partner will develop warts themselves [1,2]. There have been over one hundred types of HPV identified with around forty genotypes that are known to affect the anogenital area [3]. Though primarily (90%) HPV genotypes 6 and 11 are known to be associated with external anogenital warts, combined infections with other low-risk (LR) and high-risk (HR) HPV types also occur [4,5,6,7,8,9]. People who are between 18 and 59 years of age are most commonly affected, with the highest frequency reported for those between 20 and 35 years. In adolescents, external condyloma acuminata are the most common clinical manifestations of HPV infection and up to 83% of females with external anogenital warts or a history of external anogenital warts have concomitant cervical HPV infection [4]. It has also been indicated that patients with simultaneous infection are at higher risk of developing more serious diseases like cervical, penis, and anal cancer [6]. Besides, around 20% of people with EGW will present other sexually transmitted diseases (STDs) [10]. Considering this, patients with a diagnosis of EGW should be regarded as part of the high-risk population and optimal follow-up should be administered [6,11]. These patients also pose serious risks in the transmission of HPV and other STDs in the population. Approximately 70% of the HPV infections resolve spontaneously in one year and 90% in two years, while HPV persistence develops in the remainder and such patients require treatment [12]. Therapy options include home-based (cytodestructive and immunomodulatory therapies) and clinician-administered treatments (cryotherapy, electrotherapy, laser, and surgery). However, these interventions may solve the clinical problem, but do not eliminate the basic viral cause [13,14]. Furthermore, depending on the applied method, recurrence can be as high as 30–65%; therefore, primary prevention by HPV vaccines must be highlighted [15,16,17,18,19]. Since external anogenital warts are not a reportable disease, its incidence is difficult to estimate. Nevertheless, based on the results of systematic reviews, 9 to 13 percent of the global population is infected; the regional distribution of new cases of external anogenital warts/100,000 population/year is approximately 101 to 205 in North America, 118 to 170 in Europe, and 204 in Asia [1]. The distribution of the different HPV genotypes varies across different populations and geographical regions [20,21,22,23,24]. Data concerning the persistence of HPV genotypes among patients diagnosed with EGW, in different geographical regions are limited. Since such data are not yet available from Hungary, in a retrospective study, we aimed to determine the prevalence of HPV genotypes in patients diagnosed with EGW by using the HPV Direct Flow CHIP test, which is sensitive to thirty-five different genotypes (17 LR, 18 HR) of HPV. In our view, our data could provide invaluable information concerning the prevalence of HPV genotypes in the target population, enabling improved assessment of the actual and future efficacy of vaccination programs, vaccine development, and forecast changes in infection patterns [25,26,27,28].

## 2. Materials and Methods

### 2.1. Study Population and Sample Preparation

Archived, formalin-fixed paraffin-embedded (FFPE) samples from 94 patients (*n* = 66 females; *n* = 28 males), earlier diagnosed with EGW, were processed in our retrospective study. The patients mostly resided in the south-west part of Hungary. The study was performed based on females and males that attended the Departments of Obstetrics and Gynecology, Urology and Department of Dermatology, Venereology, and Oncodermatology at the University of Pécs, between 2004 and 2020, due to routine gynecological and urological screening, or because of symptoms of external anogenital warts, such as pain, bleeding or aesthetic reasons. Patient’s data (e.g., age at diagnosis, area of residence, date of sample collection, history of the prevalence of EGW, history of treatments, history of smoking habits, clinical pathology, the morphology of the lesions, and sampling sites) were extracted from medical records.

### 2.2. HPV Genotyping

Determination of HPV genotypes was accomplished by the HPV Direct Flow CHIP test (Master Diagnostica, Granada, Spain). The technology is based on the amplification of a short DNA fragment of HPV L1 ORF, enabling the use of FFPE samples. The test allows the qualitative detection of thirty-five different genotypes of HPV (high-risk HPV: HPV 16, 18, 26, 31, 33, 35, 39, 45, 51, 52, 53, 56, 58, 59, 66, 68, 73 and 82; and low-risk HPV: HPV 6, 11, 40, 42, 43, 44, 54, 55, 61, 62, 67, 69, 70, 71, 72, 81 and 84) based on direct PCR from crude-cell extracts, automatic flow-through hybridization and colorimetric detection. All the virus types are determined separately except for the low-risk 44/55 and 62/81 genotypes, which are identified in clusters.

The test was performed according to the manufacturer’s instructions. In brief: 3 pieces of 10 µM thick sections were treated with 400 µL of mineral oil then incubated at 95 °C for 2 min. After removing the mineral oil, 60 µL lysis buffer with 1.5 µL DNA release (Master Diagnostica, Granada, Spain) was added to the samples and incubated at 60 °C for 30 min, followed by inactivation at 98 °C for 10 min. To lyophilized PCR mix, 3 µL of crude cell extract and 27 µL DNase/RNase free water were added. The lyophilized PCR mix contained PCR buffer, dNTP(U/T), DNase/RNase free water, biotinylated primers, DNA polymerase, and UNG. The primers included are specific for the amplification of a fragment of the L1 region of the HPV genome. Besides, primers for the amplification of the human beta-globin gene are included and used as an internal control for the PCR reaction. The amplification cycling conditions in IANLONG PCR Thermal Cycler (Genesy 96T) were the following: pre-incubation at 25 °C for 10 min then 94 °C for 3 min; 15 cycles of denaturation at 94 °C for 30 s, annealing at 47 °C for 30 s and elongation at 72 °C for 30 s; 35 cycles of denaturation at 94 °C for 30 s, annealing at 65 °C for 30 s and elongation at 72 °C for 30 s and final elongation at 72 °C for 5 min; followed by cooling at 8 °C. Amplicons were denatured at 95 °C for 10 min then cooled on ice for 5 min.

**Figure 2:**
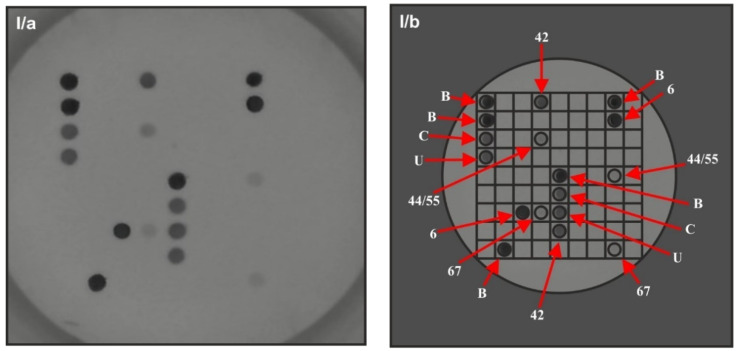

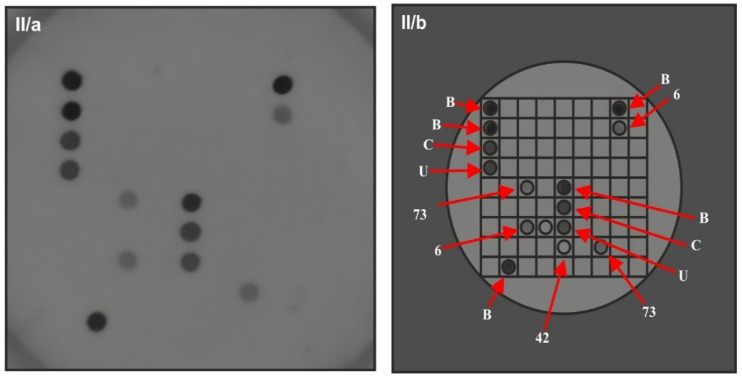
HPV genotype detection with Direct Flow CHIP test. I: Low-risk 6, 42, 44/55 and 67 HPV genotype multi-infection in patient with external anogenital warts. (**I/a**, **I/b**): snapshots before and after processing with hybriSoft software. Controls and the HPV genotypes were tested in duplicates. II: Low-risk 6 and high-risk 73 HPV genotype coinfection in patient with external anogenital warts. (**II/a**, **II/b**): snapshots before and after processing with hybriSoft software. Spot B: Hybridization control (5 signals to orientate the CHIP). Spot C: Internal DNA control (genomic human DNA probe). Spot U: HPV universal probe.

The full hybridization process was performed semi-automatically in hybriSpot (HS12) following the instructions provided by the system. The management of the samples, the capture of images, and the analysis and report of the results were performed by hybriSoft software (Figure 2).

### 2.3. Statistics

The overall prevalence of HPV genotypes was calculated. The distribution of specific HPV genotypes was expressed as the proportion of HPV DNA-positive specimens among all cases of EGW. Qualitative variables were studied using the two-sided Chi test or Fisher’s exact test as appropriate. Quantitative data are expressed as mean (±SD) and range. *p* values < 0.05 were considered to be statistically significant.

## 3. Results

### 3.1. Patients Characteristics

Samples from sixty-six females (mean age 36 ± 16.9 years; median age 29.5 years; range 15–68 years) and twenty-eight males (mean age 37.5 ± 18.5; median age 32.5 years; range 21–77 years) were processed in the study; all of them provided positive β-globin results, suggesting that the DNA amount were suitable for PCR analysis. All patients were confirmed to be HPV positive and all the genotypes had been identified. The patient characteristics are presented in Table 1. Based on medical records, among females, 30.3% of all cases were a clinically recurrent disease. We did not find an association between the incidence of clinically recurrent cases and the age at diagnosis. Data according to the type of EGW diagnosis (clinically recurrent or new) were not available among male patients. At the time of diagnosis, 9.1% of females with EGW were pregnant. The EGW lesions mostly appeared in multiple forms, 66.6% and 64.2% in the case of female and male patients, respectively. Data on smoking habits were only available among females with the exclusion of five cases; according to these, 40.9% of the females were smoking at the time of diagnosis. Comparing the twenty-seven patients who were smokers to non-smokers, we identified thirteen clinically recurrent cases and fourteen new cases in the smoker’s group. When we compared the findings with the non-smoker’s group, only six cases were found with clinical recurrence and twenty-one with a newly diagnosed lesion; the association between a smoking habit and clinically recurrent disease showed a significant relationship (*p* = 0.035, at *p* < 0.05).

### 3.2. HPV Prevalence

HPV genotype classification was based on the HPV Direct Flow CHIP test’s (Master Diagnostica, Granada, Spain) description. HPV DNA was detected in all of the ninety-four EGW cases and genotypes were also determined in every case. From the ninety-four cases, sixty-eight (72.3%) showed one HPV genotype, while in twenty-six cases (27.6%), multi-infections were found. According to our results, none of the cases occurred with high-risk genotype without a low-risk genotype, whereas a total of eighteen (19.1%) samples demonstrated coinfection with low-risk and high-risk genotypes, and 4.3% of the samples were infected with more than one high-risk HPV (Table 2). Comparing multi-infections to mono-infections in regard to mean ages, we found that although the difference was not significant, multi-infections occurred more frequently at younger ages (34.5 compared to 38.9 *p* = 0.276; at *p* < 0.05). The presence of ≥1 high-risk genotype was as common among males as females (*p* = 0.836; at *p* < 0.05) and occurred as frequently in the new cases as in the clinically recurrent cases (*p* = 0.742; at *p* < 0.05; data not significant). Our results also indicated that ≥1 high-risk genotype was mostly found at ages under thirty years (*p* = 0.142; *p* < 0.05; data not significant).

### 3.3. Occurrence of the Specific Genotypes

Out of the thirty-five tested (17 low-risk, 18 high-risk) HPV genotypes, ten low-risk, and eleven different high-risk HPV genotypes were detected, yielding a total number of one hundred thirty-one infections among the ninety-four samples (Table 3). Out of the one hundred thirty-one infections, one hundred nine were low-risk, while twenty-two were high-risk. The most frequently identified HPV genotypes were as follows, by decreasing frequency: HPV 6 (61.8%), HPV 11 (9.2%), HPV 42 (4.6%) and HPV 40, HPV 56, HPV 59, HPV 66, HPV 73 (2.3%). Out of the one hundred thirty-one infections, DNA of the low-risk HPV 6 and HPV 11, alone or in combination with other HPV genotypes, showed the highest prevalence of 71%. Correlating the prevalence of HPV 6 and HPV 11 to the number of cases, we found that 98.9% of the samples were carrying either of these. The only low-risk type that we were able to detect independently from HPV 6 and 11 in one case, was HPV 40 whereas DNA of the other low-risk HPV types including 42, 44/55, 54, 67, and 62/81 were always found in combination with HPV 6 and/or HPV 11. Low-risk genotypes, with the exception of HPV 6 and 11 represented 12.2% of all infections, yielding 14.7% of the low-risk infections. The most common high-risk HPV genotypes as HPV 56, 59, 66, and 73 were detected at the same frequency as 2.3%, yielding an overall prevalence of 9.2% of all infections, and 54.5% of the high-risk infections. The frequency of the two most studied high-risk HPV genotypes, 16 and 18 were 1.5% each of all infections, yielding 9.1% of the high-risk infections. From the detected eleven high-risk types among EGW cases, ten (90.9%) types (HPV 16, 18, 39, 51, 52, 56, 66, 68, and 73) were found among females, while four (36.46%) types (HPV 18, 53, 56 and 73) were observed among males, yielding to a 70% discrepancy between female and male patients. In contrast, excluding the three overlapping genotypes (HPV 18, 56, and 73), the difference between male and female cases was only 10%; the DNA of HPV 53 was the only one that was just observed among male EGW cases. However, the results are not significant, more types of high-risk HPV DNA were represented among females than male EGW cases (*p* = 0.46 at *p* < 0.05). In the case of the low-risk genotypes, 60% of the detected HPV types shared homology (HPV 6, 11, 40, 42, 54, 67) in female and male EGW cases, while 40% of the genotypes (HPV 44/55 and 62/81) were only found in females (Table 3).

## 4. Discussion

It has been proposed that low-risk HPV genotypes do not integrate their DNA into the chromosomes of the infected cells, hence such low-grade lesions have a low risk of progression to malignancy. However, around 19–33% of EGW cases are coinfected with oncogenic, high-risk HPV genotypes [6,22]. While most of the studies and screening programs focus on the HPV-related malignant disease, the epidemiology of external anogenital warts is not well characterized. Data regarding the prevalence of different HPV genotypes related to EGW, especially in Mid-European countries, are underrepresented and incomplete [22]. The present study aimed to determine the prevalence of different HPV genotypes in surgical external anogenital wart samples from patients diagnosed with hard-to-treat EGW. Formalin-fixed paraffin-embedded samples of ninety-four patients (sixty-six females and twenty-eight males) were recruited in the study. The significantly smaller sample size of the men’s group could be due to the different attitudes to visiting doctors. Unlike females who routinely take part in screening programs and undergo follow-up with gynecologists, males usually seek consultation with specialists if they present symptoms. Asymptomatic individuals are often not identified, representing an invisible segment for healthcare systems. Despite the relatively small sample size of our cohort, we were able to gather valuable information regarding HPV distribution. The observed overall 100% prevalence of low-risk HPV infection indicated that EGW lesions never appear in the absence of low-risk HPV infection. Based on our results we found a significant relationship between smoking habits and clinically recurrent disease (*p* = 0.035). According to our results, among the examined Hungarian patients the most common HPV genotypes were HPV 6 and 11. The vast majority of the samples, 72.3%, were diagnosed with mono-infection of HPV 6 and 11 genotypes, while these two genotypes either alone or in combination with other HPV genotypes, occurred in 98.9% of the cases. Data regarding the prevalence of HPV 6 and 11 genotypes are comparable to the data reported from other European countries [5,21,22]. By applying a highly sensitive method we were able to identify a wide spectrum of HPV coinfections [29,30,31,32]. In our study, 27.6% of the observed cases were multi-infections. There were no significant differences in the presence of multi-infections among females and males. Comparing multi-infections and mono-infections by the mean ages, although data were not significant, we could conclude that multi-infections mainly occurred at a lower mean age (34.5 vs. 38.9 years).

We found that oncogenic, high-risk HPV genotypes never occurred as mono-infection. Our study revealed, that 19.1% of the cases occurred with ≥1 high-risk HPV genotype. Although data were not significant, by analyzing the prevalence of high-risk genotypes according to different age distributions, in line with other studies, we found that ≥1 high-risk genotype was more represented in the age group of under thirty years of age [4]. This could be explained by lifestyle attributes. Furthermore, our results also indicated more HPV genotypes among female patients. Interestingly, we observed slight differences with regard to the prevalence of the most frequently appearing high-risk genotypes compared to the data reported by other European countries; instead of HPV 16 being the most common high-risk type, HPV 56, 59, 66, and 73 were the most frequent genotypes [21,22]. In the case of the other tested genotypes, the prevalence was comparable to the data reported in other European countries [5,21,22].

Based on the analysis of Mayenaux and Chaturwedi, the incidence of HPV infection could be kept in check; although the continuously increasing number of risk factors, such as the growing number of lifetime sexual partners, earlier sexual debut (<16 ages), homosexual relationships, history of other STDs, smoking and human immunodeficiency virus (HIV), can only be counteracted by screening programs and vaccination [15,33,34].

Currently, three licensed HPV vaccines are available using L1 capsid antigens of two, four, or nine HPV genotypes. All three vaccines include HPV 16 and 18, which cause the majority of HPV-related cancers; the quadrivalent vaccine (Gardasil 4) also targets low-risk types HPV 6 and 11, which are primarily associated with external anogenital warts, while the nonavalent (Gardasil 9) vaccine also includes five other high-risk HPV types such as HPV 31, 33, 45, 52, and 58. Preliminary data also indicate that vaccination against one HPV strain may, in turn, confer protection against heterologous HPVs [27,28,35]. High efficacy of the quadrivalent and nonavalent HPV vaccines against HPV 6 and 11-associated disease has been reported in multiple randomized, controlled trials [36,37,38]. There is accumulating evidence that population-based vaccination targeting both HPV 6 and 11 can result in dramatic declines in the incidence of genital warts [35,37]. Although the present study did not include data on the individual vaccination status, we found that the prevalence of HPV 6 and/or HPV 11 was 98.9% among all EGW cases, indicating a potentially great benefit from the available vaccines covering these types. However, it must be highlighted that the prevalence of other high-risk and low-risk HPV genotypes that are not targeted by direct vaccination or cross-protection remains quite high, suggesting the importance of follow-up for patients diagnosed with EGW. Out of the eleven detected high-risk HPV genotypes the nonavalent vaccine covers only three, HPV 16, 18, and 52, while out of the ten detected low-risk genotypes, it covers only two types, HPV 6 and 11. The observed four most frequent high-risk genotypes, including HPV 56, 59, 66, 73, and the rest of the low-risk genotypes are not included in any kind of available vaccine so far, yet, the affected patients represent a high risk for spreading these genotypes in the population. Considering this, our results underline the importance of developing new wider-spectrum vaccines against HPV. Recent preclinical data have indicated that the induction of cross-neutralization antibodies by modified L1 or L2 epitopes may be a possible strategy for the generation of broad-spectrum vaccines. With modifications, an improved HPV vaccine could provide protective immunity against twenty types of HPV [28,38,39]. In Hungary, the bivalent vaccine has been available since 2007 while the quadrivalent vaccine, which was available from 2006, was replaced with the nonavalent vaccine in 2015. As part of the national immunization program, in 2014 the bivalent HPV vaccine was introduced to 13-year-old girls through a school-based vaccination program. In 2018 the bivalent vaccine was replaced and since then the nonavalent vaccine has been applied, providing protection against EGW as well. The acceptance of the vaccine is increasing among girls, which was around 80% in 2019, based on the results of the State Public Health and Medical Officer. It is a great advance that males at 13 years of age have also been recruited in the national HPV vaccination program from 2020. Although the attitude seems positive, educating the population could further improve the coverage of the vaccine [40].

## 5. Conclusions

The published data on type-specific HPV-associated diseases in Central and East European countries are scarce. Our retrospective study is the first to provide data on the prevalence of different HPV genotypes among Hungarian patients diagnosed with EGW. While most of the studies have analyzed only small numbers of genotypes, our wide spectrum analysis of HPV genotypes will contribute to understanding the HPV prevalence in EGW and may have an impact on vaccine development and the HPV vaccination program in Hungary.

## 6. Limitation

Although our results show high accordance with those of other studies on HPV and EGW, the relatively small sample size and limited clinical data for male patients made the statistical comparison difficult.

## Figures and Tables

**Table 1:** Characteristics of ninety-four Hungarian patients diagnosed with external anogenital warts (EGW).

Characteristic	Value
**Sex**	
Female	66 (70.2%)
**Age, median years (range)**	36 (15–68)
Range of age groups	
15–20	17 (25.7%)
20–24	9 (13.6%)
24–30	8 (12.1%)
30–40	4 (6.0%)
40–50	9 (13.6%)
50–60	13 (19.6%)
>60	6 (9.0%)
**Pregnant at time of diagnosis (n = 66)**	
yes	6 (9.1%)
no	60 (90.9%)
**Type of EGW diagnosis**	
New diagnosis	46 (69.7%)
Clinically recurrent disease	20 (30.3%)
**Appearance of the lesions**	
Simple	22 (33.3%)
Multiple	44 (66.6%)
**Smoking**	
yes	27 (40.9%)
no	39 (59.0%)
data not available	5 (7.6%)
**Sex**	
Male	28 (29.8%)
Age, median years (range)	37.5 (21–77)
**Range of age groups**	
20–24	6 (21.4%)
24–30	6 (21.4%)
30–40	7 (25.0%)
40–50	-
50–60	3 (10.7%)
>60	6 (21.4%)
**The appearance of the lesions**	
Simple	10 (35.7%)
Multiple	18 (64.2%)

**Table 2:** Prevalence of human papillomavirus (HPV) infection in external anogenital warts (EGW), according to low-risk (LR) and high-risk (HR) classification.

No (%) of Cases				
Variable	Male	Female	Total	*p*
	(*n* = 28)	(*n* = 66)	(*n* = 94)	
**Mono-infections**	21 (75.0%)	47 (71.2%)	68 (72.3%)	0.707
LR HPV types	21 (75%)	47 (71.2%)	68 (72.3%)	0.707
HR HPV types	0	0	0	N.A.
**Multi-infections**	7 (25.0%)	19 (28.8%)	26 (27.6%)	
Only LR HPV	2 (7.1%)	6 (9.1%)	8 (8.5%)	1
Only HR HPV	0	0	0	N.A.
LR and HR HPV	5 (17.8%)	13 (19.7%)	18 (19.1%)	0.836
1 HR HPV	4 (14.3%)	10 (15.2%)	14 (14.9%)	1
>1 HR HPV	1 (3.6%)	3 (4.5%)	4 (4.3%)	1

**Table 3:** Prevalence of human papillomavirus (HPV) genotypes and infection numbers among ninety-four patients diagnosed with external anogenital warts (EGW), by decreasing order of frequency.

No (%) of Cases			
HPV Genotype	Number of Mono-Infection (68)	Number of Multi-Infection (63) (with other LR and/or HR)	Number of Total Infections (131)
**Low-risk (LR)**			
**HPV genotypes**			
HPV 6	59 (86.8%)	22 (34.9%)	81 (61.8%)
HPV 11	9 (13.2%)	3 (4.7%)	12 (9.2%)
HPV 42	-	6 (9.5%)	6 (4.6%)
HPV 40	-	3 (4.7%)	3 (2.3%)
HPV 44/55	-	2 (3.2%)	2 (1.5%)
HPV 54	-	2 (3.2%)	2 (1.5%)
HPV 67	-	2 (3.2%)	2 (1.5%)
HPV 62/81	-	1 (1.6%)	1 (0.8%)
**High-risk (HR)**			
**HPV genotypes**			
HPV 56	-	3 (4.7%)	3 (2.3%)
HPV59	-	3 (4.7%)	3 (2.3%)
HPV 66	-	3 (4.7%)	3 (2.3%)
HPV 73	-	3 (4.7%)	3 (2.3%)
HPV 16	-	2 (3.2%)	2 (1.5%)
HPV 18	-	2 (3.2%)	2 (1.5%)
HPV 51	-	2 (3.2%)	2 (1.5%)
HPV 39	-	1 (1.6%)	1 (0.8%)
HPV 52	-	1 (1.6%)	1 (0.8%)
HPV 53	-	1 (1.6%)	1 (0.8%)
HPV 68	-	1 (1.6%)	1 (0.8%)

## Data Availability

The datasets used and/or analyzed during the current study are available from the corresponding author on reasonable request.

## Abbreviations

EGW: external anogenital warts; FFPE: formalin-fixed paraffin-embedded; HR: high-risk; HIV: human immunodeficiency virus; HPV: human papillomavirus; LR: low-risk; STD: sexually transmitted disease
